# Black Chokeberry Fruit Polyphenols: A Valuable Addition to Reduce Lipid Oxidation of Muffins Containing Xylitol

**DOI:** 10.3390/antiox9050394

**Published:** 2020-05-07

**Authors:** Jaroslawa Rutkowska, Agata Antoniewska, Montserrat Martinez-Pineda, Agnieszka Nawirska-Olszańska, Anna Zbikowska, Damian Baranowski

**Affiliations:** 1 Institute of Human Nutrition Sciences, Faculty of Human Nutrition, Warsaw University of Life Sciences (WULS-SGGW), Nowoursynowska st.159c, 02-776 Warsaw, Poland; agata_antoniewska@sggw.pl (A.A.); damian_baranowski@sggw.pl (D.B.); 2 Department of Animal Production and Food Science, Faculty of Sports and Health Science, University of Zaragoza, Plaza Universidad no. 3, 22002 Huesca, Spain; mmpineda@unizar.es; 3 Department of Fruit, Vegetable and Plant Nutraceutical Technology, Wrocław University of Environmental and Life Sciences, Chełmońskiego 37, 51-630 Wrocław, Poland; agnieszka.nawirska-olszanska@upwr.edu.pl; 4 Institute of Food Sciences, Department of Food Technology and Assessment, Division of Fat and Oils and Food Concentrates Technology, Warsaw University of Life Sciences (WULS-SGGW), Nowoursynowska st.159c, 02-776 Warsaw, Poland; anna_zbikowska@sggw.pl

**Keywords:** chokeberry polyphenol extract, antioxidative properties, hydroperoxides, secondary lipid oxidation products, fatty acids, muffins, xylitol

## Abstract

The study aimed at assessing effects of black chokeberry polyphenol extract (ChPE) added (0.025–0.075%) to xylitol-containing muffins to reduce lipid oxidation, especially in preventing degradation of hydroperoxides throughout the storage period. Among polyphenolic compounds (3092 mg/100 g in total) in ChPE, polymeric procyanidins were the most abundant (1564 mg/100 g). ChPE addition resulted in a significantly increased capacity of scavenging free radicals and markedly inhibited hydroperoxides decomposition, as reflected by low anisidine values (AnV: 3.25–7.52) throughout the storage. On the other hand, sucrose-containing muffins had increased amounts of primary lipid oxidation products and differed significantly from other samples in conjugated diene hydroperoxides (CD values), which was in accordance with the decrease of C18:2 *9c12c* in those muffins after storage. In addition, sucrose-containing muffins were found to be those with the highest level of contamination with toxic carbonyl lipid oxidation products. Throughout the storage, no yeast or moulds contamination were found in higher enriched muffins. The incorporation of polyphenols to xylitol-containing muffins resulted in preventing decomposition of polyunsaturated fatty acids (PUFAs), and in reducing the content of some toxic aldehydes. ChPE could be regarded as a possible solution to xylitol-containing muffins to extend their shelf life. The results support the use of xylitol in muffin manufacture as being favourable in terms of suitability for diabetics.

## 1. Introduction

Consumers appreciate muffins, but those that contain sucrose, high-fructose syrup or invert sucrose have a high glycemic index and are not recommended for diabetics. Moreover, high-fructose syrups, containing 42–55% fructose, are considered more lipogenic than sucrose, thus more risky for non-alcoholic fatty liver disease and dyslipidemia [1]. Xylitol, used in this study instead of high-fructose syrups or sucrose is, like the majority of other polyols, only slowly absorbed from the digestive tract, largely due to the lack of a specific absorption system in the intestinal mucosa; only 25–50% of the ingested xylitol is absorbed from the intestine. The conversion of xylitol to glucose in the liver is very slow and does not increase blood glucose level to a significant extent [2]. Studies of Shim et al. [3] revealed that intestinal uptake of total catechins significantly increased 6 or 11 times in green tea with xylitol/citric acid or xylitol/vitamin C, respectively, compared to unsweetened green tea. However, the use of polyols in bakery products remains underappreciated.

Another weak point of baked confectionery manufacture is the use of synthetic antioxidants—butylated hydroxyl anisole (BHA) and butylated hydroxyl toluene (BHT), to keep products fresh for longer periods. Although using BHT is considered generally safe as food preservative when used at the approved content, there is debate whether BHT exposure is linked to cancer, asthma, and behavioral issues in children [4]. Because of that, in 1987, the EU Scientific Committee for Food established acceptable daily intake (ADI) of BHT of 0.05 mg/kg bw/day [5]. Although European Food Safety Authority (EFSA) *Panel on Food Additives and Nutrient Sources added to Food* in 2012 concluded that the present database gives reason to revise the ADI of BHT of 0.05 mg/kg bw/day, it is still remained unchanged [5]. In their scientific opinion, the EFSA also noted the exceeding ADI of BHT by children in some European countries (Finland, The Netherlands) at the 95^th^ percentile [5]. In 2011 EFSA *Panel on Food Additives and Nutrient Sources added to Food* concluded that ADI of BHA of 0.5 mg/kg bw/day may be revise [6]. In 2018, EFSA delivered a scientific opinion that no concern for consumer safety would arise from the use of BHA as a feed additive at the maximum concentrations of 150 mg/kg feed [7]. However, EFSA stated that allergy or intolerance of orally administered BHA is unknown [6].

This calls for “clean label” products, both nutritious and low in calories. In effect, a number of studies were devoted to the use of polyphenol-rich plants, e.g., raspberry and cranberry pomace powder, cherry pomace extract encapsulated in whey and soy proteins, Japanese quince fruits, and tea leaves as natural antioxidants in bakery products [8,9,10,11].

Black chokeberry (*Aronia melanocarpa* [Michx] Elliot, *Rosaceae*) fruits contain an extraordinary number of polyphenolic compounds in high levels: proanthocyanidins, anthocyanins and phenolic acids, which have distinctive antioxidant properties. Proanthocyanidins (oligomeric and polymeric (epi) catechins), are the dominant phenolic compounds in chokeberry fruits, with their content reaching 5% of whole fruit dry mass [12,13]. Anthocyanins represent about 25% of total chokeberry phenols and include cyanidin 3-glucoside, 3-galactoside, 3-xyloside, 3-arabinoside, pelargonidine-3-galactoside and pelargonidine-3-arabinoside [13,14,15]. There is strong evidence supporting health benefits of black chokeberry. Sidor, Drożdżyńska and Gramza-Michałowska [14] summarized the beneficial effects of black chokeberries in frequent co-morbidities such as dyslipidaemia, hypertension, obesity, glucose metabolism disorders, pro-inflammatory conditions, and thrombosis risk. Also, Sikora et al. [16] indicated that diets enriched with black chokeberry juice or extract improved the lipid balance of subjects with metabolic syndrome. Black chokeberry also revealed potential to inhibit the development of various types of cancers, including leukaemia, breast and intestinal cancer. Anti-proliferative and protective effects of chokeberry extract against colon cancer were confirmed by in vitro studies of Malik et al. [17].

Previous studies revealed that thermal processing affects the stability and properties of fruit polyphenols [10,18,19,20]. Among polyphenols, anthocyanins are the most sensitive to thermal treatment [21]. Górnaś et al. [19] demonstrated that anthocyanins from strawberry, black currant and raspberry in muffins, differed greatly in thermal stability (36–97% lost); in contrast the flavonol glycosides were the most stable (0–21% lost). Baking increases the amount of free phenolic acids in bread, cookies and muffins but decreases that of bound phenolic acids [10,22]. Although bakery products are contaminated with lipid oxidation compounds derived from heat-treated fats, the problem is still underappreciated by many authors. Systematic review of Ou et al. [21] pointed that toxic aldehydes were detected in bakery products. Another problem is the appearance of hydrocarbons, also aromatic, as contaminants of cookies [8]. Niki [23] pointed that α, β-unsaturated aldehydes were highly reactive and readily reacted with proteins, DNA, and phospholipids, producing deleterious effects. It was also reported that lipid hydroperoxides were not harmless for human health, as they were elevated in patients suffering from e.g., hyperlipidemia, atherosclerosis, diabetes mellitus, stroke, and multiple sclerosis. The review of Ou et al. [21] suggested that the incorporation of polyphenols reduced the content of some toxic aldehydes in baked foods and scavenged toxic aldehydes in thermal-treated food. Previous results indicated about limitations in the content of chokeberry fruit extract Aronox (Agropharm,Tuszyn, Poland). Enrichment of cookies with 0.090% amount of this chokeberry extract revealed as having prooxidative properties and attributed to high intensity sensory astringent taste, acidic taste, off-taste and off-flavour [24]. We thus assumed that chokeberry fruit extract, rich in polyphenols, might be effective as an antioxidative agent for reducing lipid oxidation in muffins in lower than previously studied amounts. The aim of this study was thus to examine the effects of supplementing the xylitol-containing muffins with chokeberry fruit extract (in range of 0.025%, 0.05% and 0.075%) on reducing the lipid oxidation process, especially in preventing the degradation of hydroperoxides throughout storage, as well as on the acceptability by potential consumers.

## 2. Materials and Methods

### 2.1. Chokeberry Polyphenol Extract (ChPE)

Ripe black chokeberry fruits were manually collected at a plantation located on the slopes of the Eagle Mountains (southwestern Poland) in August 2017, at the fully ripened stage. The berries were washed with distilled water, surface-cleaned with cotton cloth and then dried in a laboratory dryer at 40 °C for 48 h. Dried berries were ground using an electric grinder to obtain particles measuring about 300 μm.

Polyphenol extract from dried chokeberry fruits powder was prepared by macerating berries in an Erlenmeyer flask (100 mL) on a shaker (Unimax 1010, Heidolph, Germany) at ambient temperature, and then extracted with 50% ethanol for 60 min, the solid-to-solvent ratio being 1:20. The flasks were covered with aluminum foil to avoid light exposure and ethanol evaporation. The extracts were separated by paper filtering (POCH, S.A., Poland). These extraction conditions were previously recommended by Čujić et al. [25] for the extraction of polyphenols from chokeberry. Next, the ethanol was evaporated in a rotary vacuum evaporator (RVO 400, INGOS s.r.o., Czech Republic) and the obtained extract was frozen (−80 °C, 60 min) and freeze-dried using Christ Alpha 1-2 LD plus lyophilizer (Martin Christ Gefriertrocknungsanlagen GmbH, Germany).

### 2.2. Muffin Preparation

To stabilize the extract in dough, ChPE, prior to adding, was dissolved in 8 mL of 1.59% citric acid solution. In addition to the four variants presented in Table 1, another one was prepared, in which the xylitol was replaced by sucrose and no ChPE was added. Muffins were prepared and baked as previously reported by Białek, Rutkowska, Adamska, and Bajdalow [26].

After cooling, 24 h from baking, muffins were packed in cellulose film, put into cardboard box without light access. Muffins were stored at 23 ± 1 °C until counts of moulds exceed 10 colony forming units accordingly PN-ISO 21527-2 (2009) [27].

Baked muffins were assayed as follows: immediately after production, after 2, 4, 6, and 8 weeks of storage (with regard to antioxidant properties, lipid oxidation products and fatty acid contents). Microbiological analysis was provided in nine intervals (0–8 weeks). 

### 2.3. Chromatographic Analysis of Polyphenol Compounds in ChPE 

Polyphenols were extracted from ChPE with 30% methanol acidified with 1% acetic acid and containing ascorbic acid (1%), the mixture was incubated for 20 min under sonication (Sonic 6D, Polsonic, Warsaw, Poland). Polyphenol analysis was done using an ACQUITY Ultra Performance LC system (UPLC) equipped with photodiode array detector (PAD) and binary solvent manager (Waters Corporation, Milford, USA), coupled with quadrupole time-of-flight micro-mass spectrometer (MS; Waters, Manchester, UK) and with an electrospray ionization source operating in negative and positive modes. For separation, a UPLC BEH C18 column was used (1.7 μm, 2.1 mm × 50 mm, Waters Corporation, Milford, MA, USA). The mobile phase consisted of aqueous 0.1% formic acid and 100% acetonitrile. Samples (10 μl) were eluted according to the linear gradient according to Kolniak-Ostek [28]. Detection wavelengths were set at 280 nm (flavan-3-ols and hydroquinones), 320 nm (phenolic acids), 340 nm (flavones), 360 nm (flavonols) and 520 nm (anthocyanins). The conditions of MS were: source block temperature −130 °C, desolvation temperature −350 °C, capillary voltage −2.5 kV, cone voltage −30 V and the desolvation gas (nitrogen) flow rate −300 l/h. Calibration curves were prepared according to Kolniak-Ostek [28]. All assays were done in triplicate. The results were expressed in milligrams per 100 g of ChPE.

### 2.4. Antioxidative Properties of Muffins

To evaluate changes in antioxidant activity throughout the storage, ethanolic extracts of muffins were prepared by suspending 1 g of crushed sample in 10 mL of ethanol and left for 24 h without light access. Filter paper (POCH, S.A., Poland) was used to separate extracts.

Radical scavenging activity of those extracts on 1,1-diphenyl-2-picrylhydrazyl radicals (DPPH) was analyzed according to the method of Sánchez-Moreno, Larrauri, and Saura-Calixto [29] modified by Antoniewska, Rutkowska, Martinez-Pineda, and Adamska [30]. The absorbance decrease at 515 nm in the presence of free radicals was determined using UV-Vis spectrophotometer (Specord 40, Analytik Jena AG, Germany).

The ABTS^+^ (2,2′-azinobis-(3-ethylbenzothiazoline-6-sulfonic acid) radical cation) scavenging activity of muffins was measured spectrophotometrically according to modified method of Re et al. [31] and Serpen, Gökmen, Pellegrini, and Fogliano [32]. Briefly, to obtain ABTS^+^, 5 mL of ABTS stock solution (7 mM) and 5 mL of potassium persulfate solution (2.45 mM) were left for 16 h in the dark. Then, ABTS^+^ solution was diluted with ethanol (A_734_ = 0.700 ± 0.02). An aliquot of 750 µL of muffin extract was mixed with 3 mL of ABTS^+^ solution, next samples were vortexed and the absorbance (λ = 734 nm) was measured 6 min later. The antioxidant activity (DPPH and ABTS^+^) was expressed in mmol of butylated hydroxyl toluene (BHT) per 1 g of sample by a calibration curve.

### 2.5. Fatty Acid Composition of fat Extracted from Muffins

Lipids from muffins were extracted according to Folch, Less, and Sloane-Stanley [33], using a 2:1 chloroform/methanol (v/v) mixture. Fatty acid (FA) methyl esters (FAME) were prepared by transmethylation of fat samples using H_2_SO_4_ (95%) and methanol accordingly American Oil Chemists’ Society (AOCS, 2000) [34]. FAMEs were analysed by gas chromatography (GC) using an Agilent 6890N (USA) instrument equipped with flame ionisation detector (FID). FAMEs were separated on a capillary column Rtx 2330 with highly polar stationary phase (100 m × 0.25 mm I.D. × 0.1 µm thickness, Restek Corp., USA). The operating conditions and separation of FA methyl esters (FAME) were published in detail elsewhere [35]. The Supelco 37 standard (No. 47885-U, Sigma Aldrich) was applied to identify FAs. The FAs were quantified in relation to the internal standard, nonadecanoic FA (C19:0) (Sigma, Aldrich, USA) that was added before transesterification to lipid samples.

### 2.6. Determination of Lipid Oxidation Products in Muffins

Stability of the extracted lipid fraction of muffins was followed periodically at two-week intervals during storage. The content of primary lipid oxidation products expressed as hydroperoxide value (PV; mEq O/kg of fat) was determined according to the standard titration method [36]. The anisidine value (AnV) spectrophotometric assay reflected the content of secondary lipid oxidation products and was based on the reaction of carbonyl compounds with *p*-anisidine reagent and measurement of the yellow complex at 350 nm [37]. Additionally, the contents of conjugated dienes (CD) and conjugated trienes (CT) were determined spectrophotometrically according to Pegg [38] at 233 nm and 268 nm wavelength using ultraviolet-visible (UV-Vis) spectrophotometer (Specord 40, Analytic Jena AG, Germany) as reported [30].

### 2.7. Consumer Acceptance Evaluation of Muffins

The overall consumer’s degree of liking muffins was estimated using a 9-point structured hedonic scale ranging from “like extremely - 9” to “dislike extremely - 1”. The young consumer panel (faculty students aged 19–25 years) consisted of 50 male and 130 female subjects; they were trained in the testing procedure. The evaluation of muffins was performed 24 h from baking (fresh) and in two intervals during storage. Microbiological safety was a limitation for offering consumer panel stored muffins for evaluation. In all sessions, the same members tested all types of muffins. Muffins were delivered in coded plastic containers in a random order. Panellists were provided with spring water to cleanse the palate between tasted samples.

### 2.8. Microbiological Analysis of Muffins

The numbers of yeasts and moulds colonies were determined according to PN-ISO 21527-2 [27] presented in detail by Antoniewska et al. [30].

### 2.9. Data Analysis 

All assays were run in triplicates, the results for each sample being expressed as mean ± standard deviation (SD). The effects of ChPE content and storage time on antioxidant activity and formation of lipid oxidation products in muffins and hedonic consumer acceptability were assessed by two-way ANOVA followed by Tukey’s post-hoc test. Also, the effects of ChPE content and storage time on antioxidant activity of muffins were analyzed with regression analysis. In multiple linear regressions, the dependent variable (y) was ABTS^+^ or DPPH, the independent ones were the time of storage and ChPE content. The results of fatty acid contents were subjected to one-way ANOVA followed by Tukey’s post-hoc test. The level of p < 0.05 was considered significant. Statistical analyses were performed with GraphPad Prism 6 (GraphPad Software, Inc., San Diego, CA). 

## 3. Results and Discussion

### 3.1. Polyphenol Composition of Chokeberry Extract (ChPE)

Application of the ultra-performance liquid chromatography coupled to photodiode array detection and mass spectrometry (UPLC/PDA/MS) technique enabled determining the contents of 21 phenolic compounds in ChPE belonging to 5 groups (total content 3092 mg/100g ChPE). Among polyphenolic compounds, polymeric procyanidins were most abundant (1564 mg/100g ChPE) compared with anthocyanins, phenolic acids, flavonols and flavonones (Table 2), as reported by Oszmiański and Lachowicz [13]. However, those authors found much higher content of polymeric procyanidins than in this study; this might have resulted from using sonication by Oszmiański and Lachowicz [13] during extraction procedure, whereas in this study maceration was used.

As reported by Jovanović et al. [39], a much higher content of phenolic compounds was found when *Thymus serpyllum* herb was subjected to ultrasound-assisted fast extraction than when maceration was used. Those differences might have been also due to differences in chokeberry cultivation conditions and location, ripening stage, or in using different solvents [13,15].

As mentioned above, ChPE contained substantial amounts of polymeric procyanidins (about 50%) responsible for the pungent taste of chokeberry, and of anthocyanins, which made the dark blue colour of fruits (about 30%). Among anthocyanins, 7 compounds were separated, including the dominating cyanidin-3-O-galactoside, like reported by Oszmiański and Lachowicz [13]. However, in chokeberry cultivated in Slovenia, also small amounts of pelargonidines were detected [15].

Polymeric procyanidins, as well as anthocyanins, were reported to exhibit a variety of physiological activities, e.g., antioxidant properties, more effective than resveratrol or ascorbic acid in scavenging free radicals [40]. Their most impressive biological activities are: anti-inflammatory, antiplatelet, antimicrobial, hepatoprotective, gastroprotective, antiviral and others. Latest in vitro and ex vivo studies confirmed beneficial effect of procyanidins in cocoa bean and husk extract on vascular related dysfunction [41]. Anthocyanin-rich chokeberry extract is capable to protect endothelial progenitor cells against angiotensin II induced dysfunction [14].

### 3.2. Antioxidant Properties and Microbiological Safety of Muffins

**Figure 1:**
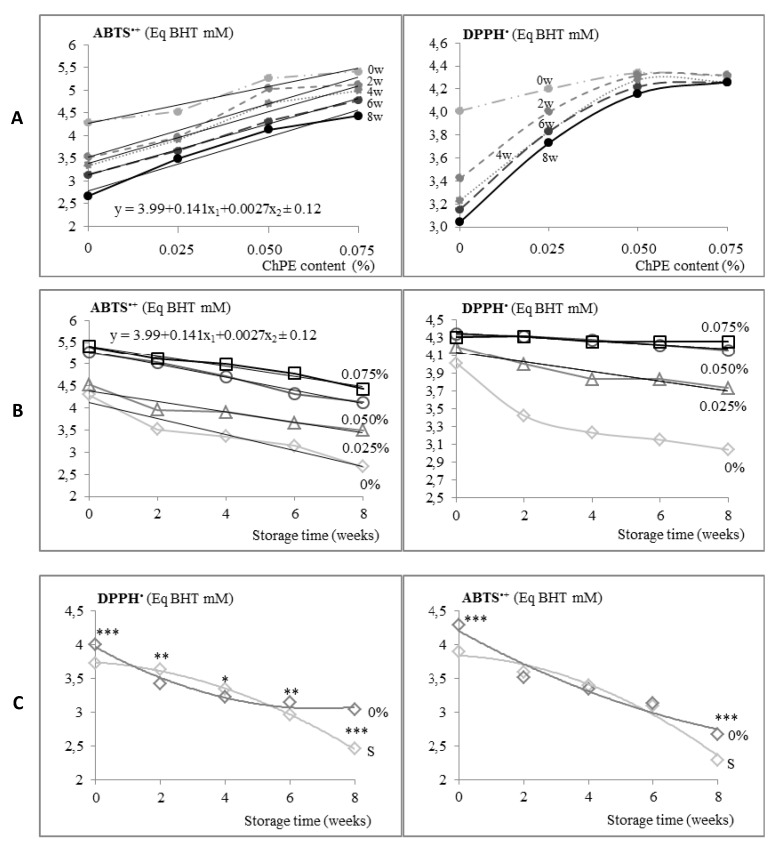
Antioxidative potential of muffins differing in ChPE (chokeberry polyphenol extract content); 0–0.075% (**A**,**B**) and in muffins containing sucrose (S) (**C**) throughout 8 weeks of storage. 0w–0-weeks of storage; 2w–2-weeks of storage; 4w–4-weeks of storage; 6w–6-weeks of storage; 8w–8-weeks of storage.

Antioxidant properties of muffins significantly (*p* < 0.05) depended on both, ChPE content (x_2_) and the time of storage (x_1_), as reflected by multiple linear regressions (see Figure 1A,B). The enrichment of muffins with ChPE resulted in significantly (*p* < 0.05) increased capacity of scavenging free radicals as reflected by DPPH and ABTS^+^ values (Figure 1A,B). Other studies also revealed that bakery products enriched with ingredients derived from fruit (pomace, by-products) like berries or sour cherry, improved its antioxidant potential [7,16].

Addition of ChPE to muffins (0.025 to 0.075%) resulted in 5–25% increase of ABTS^+^ and 5–10% of DPPH, as compared with control samples. Higher level of incorporation of ChPE into muffin formulation resulted in improving the antioxidant activity of the final product, like when using sour cherry pomace extract, freeze-dried Japanese quince fruit [8,11]. It was due to a high amount of strongly antioxidant anthocyanins and procyanidins in chokeberry extract [42]. Although heat processing brought about losses of phenolic compounds, especially anthocyanins, due to their heat instability [20], the citric acid used to dissolve the extract in muffin preparation could have acted as a protective agent, retarding the losses of this compounds during baking [43,44]. Moreover, it was demonstrated that the degree of degradation depended on the type of phenolic compound, as well as on the thermal conditions applied. Górnaś et al. [19] observed that in muffins enriched with the pomace of various berries, baked at similar conditions as ours (180 °C/18 min), anthocyanins presented higher stability than when exposed to lower temperatures at longer time (140 °C/35 min). Also, substantial amount of phenolic acids in ChPE contributed to high antioxidant properties of muffins. As previously reported, in fat-rich bakery products, phenolic acids and their derivatives were less accessible and thus less degradable than in low-fat bread products [22]. Other authors reported also increased levels of phenolic acid in enriched muffins during baking. It ought to be remembered that phenolic acids are strongly linked to cell walls, so the baking process might release them [10].

Muffins enriched with chokeberry extract kept their high antioxidant potential throughout the 8 weeks of storage (Figure 1B). As can be seen, sucrose addition significantly (*p* < 0.05) accelerated the storage time-related reduction of ABTS^+^ and DPPH contents in muffins, as compared with xylitol addition (Figure 1C). It was previously reported that over 50% of anthocyanins in blueberry puree were lost after 6-month storage. However, monomeric anthocyanins could have polymerized, these new polymeric compounds also revealed antioxidant activity [18]. The presence of xylitol in muffin formulation could be also a determinant factor. In food matrix rich in phenolic compounds, xylitol showed a protective effect on the polyphenol content, especially on anthocyanins and their antioxidant activity during storage, as previously reported by Nowicka and Wojdyło [45]. Moreover, the presence of xylitol seemed to enhance stability and to increase the intestinal bioavailability of phenolic compounds [3]. Probably because of that, the inclusion of ChPE into muffins resulted in much lower losses of scavenging capacity during storage than in control samples, as reflected by ABTS^+^ (18–22% in enriched muffins, 41% in sucrose containing ones, and 38% xylitol control muffins) and DPPH levels (2–11%, 34% and 24%, respectively; Figure 1).

Throughout the 8 weeks of storage, no yeast or moulds with counts exceeding 10 colony forming units (CFU/g) were found in muffins containing 0.05% or 0.075% ChPE, and up to 6 weeks of storage—in muffins containing 0.025% ChPE. An increased level of moulds (4.5 × 10^4^ CFU/g) was found in control muffins stored for 4 weeks, while muffins containing sucrose stored for only 2 weeks were markedly contaminated (8.5 × 10^4^ CFU/g; Table 3). Our study also confirmed antimicrobial activities of black chokeberry polyphenols like reported by Denev et al. [12].

### 3.3. Oxidative Stability of Muffins during Storage 

**Figure 2:**
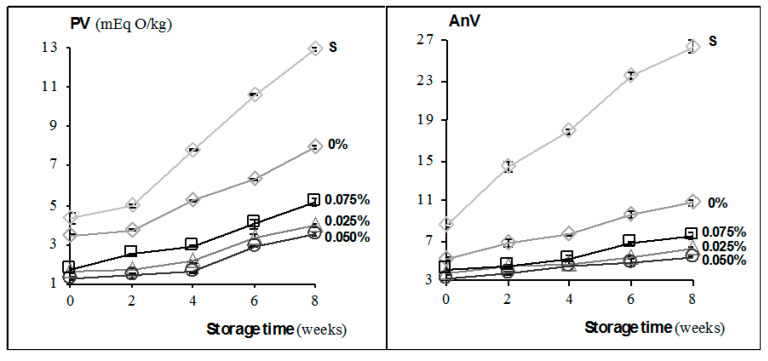
Hydroperoxide value (PV; mEq O/kg fat) and anisidine value (AnV) in muffins differing in ChPE (chokeberry polyphenol extract) content (0–0.075%) and in muffins containing sucrose (S) during storage (0–8 weeks).

Oxidative stability of fat extracted from muffins, expressed as PV-measured hydroperoxides, was significantly (*p* ˂ 0.05) related to the ChPE content and storage time (Figure 2). As compared with control and sucrose-containing samples, the enriched muffins contained lower amounts of hydroperoxides, irrespectively of ChPE amount. This was due to the strong radical scavenging activity of polyphenolic compounds, mainly polymeric procyanidins and phenolic acids in ChPE, that are more stable than anthocyanins [10], as supported also by ABTS^+^ assay of muffins (Figure 1). Literature data indicated also that procyanidins were more effective than resveratrol or ascorbic acid in scavenging free radicals [40]. Our results also support the view that plant components rich in phenolic compounds, added to cookies, are efficient antioxidants in reducing lipid oxidation [11,30,46].

**Figure 3:**
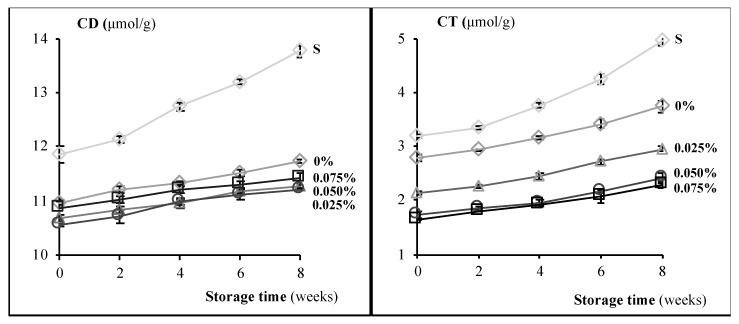
Conjugated diene (CD; μmol/g fat) and conjugated triene (CT; μmol/g fat) content in muffins differing in ChPE (chokeberry polyphenol extract) content (0–0.075%) and in muffins containing sucrose (S) during storage (0–8 weeks).

Because of that, C18:2 *9c12c* was the dominating PUFA in fat extracted from muffins (Table 4), and the majority of primary lipid oxidation products were conjugated diene hydroperoxides. Only sucrose-containing muffins differed significantly (*p* < 0.05) from other samples in CD values, accompanying the decrease of C18:2 *9c12c* in those muffins after storage (Table 4, Figure 3). As compared with literature data, the levels of CD in enriched muffins (below 10.9 μmol/g) were much lower than in muffins partially substituted with buckwheat flakes and amaranth flour blend (˂24.30 μmol/g, [30]). It should be also noted that the addition of ChPE to muffins was very low (0.025–0.075%). The levels of CT in enriched muffins were lower than the control or sucrose-containing samples, and remained relatively constant throughout the storage, while in sucrose-containing samples sharply increased after 6 weeks of storage (Figure 3). This was due to the generation of primary lipid oxidation products and to a significant decomposition of α-linolenic acid C18:3 *9c12c15c* (Table 4, Figure 2). Our results support the view that polyphenols are effective antioxidants that prevent PUFAs decomposition. Aldehydes arising from the degradation of hydroperoxides, as lipid oxidation secondary products, were spectrophotometrically assayed in fat extracted from muffins using the AnV method. The results highlight the potential of ChPE in inhibiting the degradation of hydroperoxides and in forming secondary oxidation products, irrespectively of the ChPE level. AnVs of enriched muffins ranged from 3.25 to 7.52 (Figure 2) throughout the storage and did not exceed the recommended upper limit of secondary lipid oxidation products (AnV ˂ 8), except control muffins in which it was exceeded after six week of storage, and except sucrose-containing muffins, the highest contaminated ones with toxic carbonyl lipid oxidation products (AnV ranging from 8.23 to 26.35). It was in agreement with the view that incorporation of polyphenols reduced the content of some toxic aldehydes in baked foods [21,47].

Using xylitol instead of simple sugars in muffins eliminated formation of other toxic carbonyl compounds, such as glyoxal or methylglyoxal [21]. That finding was confirmed by much lower levels of AnV in muffins containing xylitol, than in sucrose-containing samples (Figure 2). It could be concluded that using both, ChPE and xylitol in muffin formulation, not only improved their antioxidant potential, but also enhanced their safety.

### 3.4. Consumer Evaluation of Muffins

Hedonic acceptability of muffins by young consumers was significantly (*p* ˂ 0.05) related to ChPE content in the formulation and storage time (Table 5). Fresh muffins contained only 0.025% of ChPE were most appreciated in terms of overall acceptability, like the control samples. Hedonic acceptability sharply decreased at highest level of ChPE (0.075%), which resulted in the highest presence of polymeric procyanidins that probably brought about unacceptable taste of muffins. Polymeric procyanidins may impart a specific astringent and bitter taste in enriched products [13,24]. Substantial amounts of dark blue anthocyanins present in ChPE affected the colour of enriched muffins. Baking process significantly affected the anthocyanin stability in muffins and led to their degradation, as they were less stable than the colourless phenolic compounds [19]. However, higher scavenging capacity (ABTS^+^ and DPPH) of enriched muffins compared with control samples (Figure 1A,B) indicated that the thermal degradation of anthocyanins was probably limited.

Storage significantly (*p* ˂ 0.05) influenced hedonic consumer acceptability of muffins except sucrose muffins, which were not evaluated during storage because of microbiological contamination after 2 weeks storage (Table 3 and Table 5). Because of this, the microbiological safety is at a limitation for conducting consumer estimation of muffins after storage, for evaluation 3 weeks stored muffins were presented.

**Figure 4:**
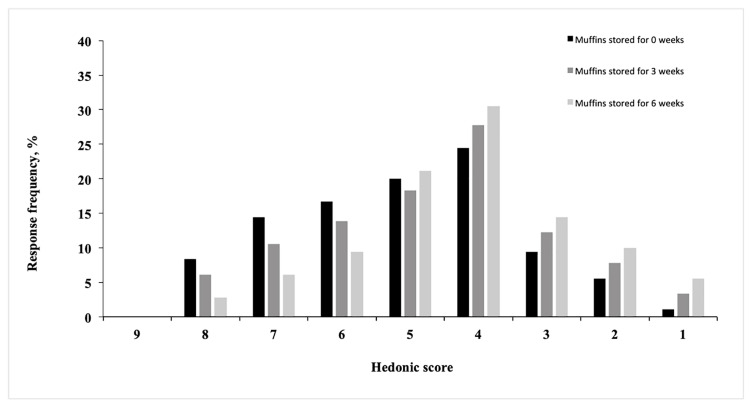
Distribution of individual preferences of muffins containing 0.075% of ChPE (chokeberry polyphenol extract): fresh muffins (0 weeks) compared with those stored for 3 and 6 weeks.

In all cases, hedonic acceptability of muffins stored for 3 weeks decreased as compared with fresh samples. In general, the consumer panel indicated a higher preference for stored muffins contained 0.025% and 0.05% ChPE. Also, in the case of enriched muffins with 0.025% and 0.05% ChPE, the decrease of acceptability after 3 weeks of storage was lower than in control muffins. As the hedonic acceptance of highest enriched muffins is limited, distribution of individual preferences during three intervals of storage (0, 3, and 6 weeks) is presented on Figure 4. Throughout storage increased number of consumers described their preferences for muffins with 0.075% ChPE as: from “dislike a little – 4” to “dislike extremely – 1”.

## 4. Conclusions

Enrichment of muffins with black chokeberry polyphenols extract (ChPE) significantly improved their antioxidative potential (ABTS^+^ and DPPH values), effectively inhibiting hydroperoxides decomposition (muffins containing 0.025% or 0.05% of ChPE had the lowest AnV) throughout the eight-week storage period. Thus, the richness of polyphenolic compounds, mainly proanthocyanidins and anthocyanins in ChPE, protected the lipid fraction of muffins with regard to the generation of toxic secondary lipid oxidation products (limitation AnV < 8). Moreover, the inclusion of ChPE in muffins might replace synthetic antioxidants used to prolong their shelf-life. Furthermore, the results represent an important approach to using xylitol and 0.025% or 0.05% ChPE in muffins in view of fulfilling the wishes of potential consumers and diabetics.

## Figures and Tables

**Table 1:** Muffin formulation.

Ingredients	Amount
Powdered Xylitol, g	100
Chokeberry polyphenol extract (ChPE), mg	0, 200, 400 or 600 mg
Refined wheat flour (WF), g	300
Egg, g	180
Milk (3.2% fat), g	120
Margarine, g	90
Baking powder, g	5

ChPE contents corresponded to 0 (control sample), 0.025, 0.05 and 0.075% per total dough mass.

**Table 2:** Polyphenol composition (means ± SD from triple measurements) in chokeberry extract (mg/100 g ChPE).

Compounds	Content, mg/100 g
*Anthocyanin, total*	823.5 ± 4.8
Cyanidin-3-pentoside-(epi)catechin	12.67 ± 0.06
Cyanidin-3-hexoside-(epi)cat-(epi)cat	10.31 ± 0.05
Cyanidin-3-*O*-galactoside	557.7 ± 4.43
Cyanidin-3-*O*-glucoside	14.09 ± 0.14
Cyanidin-3-*O*-arabinoside	205.1 ± 1.72
Cyanidin-3-*O*-xyloside	18.98 ± 0.10
Cyanidin	4.63 ± 0.04
*Phenolic acid, total*	496.8 ± 2.7
Neochlorogenic acid	174.9 ± 1.65
3-*O*-*p*-Coumaroylquinic acid	3.12 ± 0.02
Chlorogenic acid	302.8 ± 1.98
Cryptochlorogenic acid	15.98 ± 0.25
*Flavonol, total*	44.14 ± 0.32
Quercetin-dihexoside	6.04 ± 0.06
Quercetin-3-*O*-vicianoside	7.43 ± 0.08
Quercetin-3-*O*-rutinoside	7.24 ± 0.07
Quercetin-3-*O*-galactoside	13.78 ± 0.12
Quercetin-3-*O*-glucoside	9.65 ± 0.08
*Flavan-3-ols, total*	1704 ± 5
Procyanidin B2	54.23 ± 0.68
(+)-Catechin	61.64 ± 0.50
(-)-Epicatechin	23.67 ± 0.12
Polymeric procyanidins	1564 ± 5
*Flavonones, total*	23.89 ± 0.25
Eriodictyol-7-*O-*glucuronide	23.89 ± 0.25

**Table 3:** Results of microbiological analysis of muffins, fresh and stored.

Time of Storage, Weeks	Microbiological Quality of Muffins, Mould & Yeast, CFU/g *				
	Sucrose	Xylitol (0%)	X + 0.025% ChPE	X + 0.05% ChPE	X + 0.075% ChPE
0 (fresh)	<10				
1	<10	<10	<10	<10	
2	8.5 × 10^4^				
3					
4		4.5 × 10^4^ (moulds)			
5					
6					
7			2.8 x 10^4^		
8					

* - CFU/g – Colony Forming Units; ChPE – Chokeberry polyphenol extract.

**Table 4:** The content of main fatty acids (g/100 g FA) in fat extracted from muffins, fresh or stored for 8 weeks.

FA	Sucrose		Xylitol (0%)		Xylitol + 0.025% ChPE		Xylitol + 0.05% ChPE		Xylitol + 0.075% ChPE	
	Fresh	Stored 8 Weeks	Fresh	Stored 8 Weeks	Fresh	Stored 8 Weeks	Fresh	Stored 8 Weeks	Fresh	Stored 8 Weeks
C12:0	8.42 ± 1.60	7.21 ± 0.27	7.95 ± 0.05	7.38 ± 0.07	7.63 ± 0.57	8.07 ± 0.59	8.79 ± 1.05	7.66 ± 0.34	8.31 ± 0.52	8.23 ± 0.40
C14:0	3.56 ± 0.43	3.22 ± 0.14	3.43 ± 0.06	3.25 ± 0.07	3.29 ± 0.18	3.49 ± 0.23	3.86 ± 0.51	3.43 ± 0.16	3.51 ± 0.16	3.47 ± 0.06
C16:0	28.02 ± 0.76	28.82 ± 0.74	28.37 ± 0.16	28.22 ± 0.50	27.69 ± 0.54	28.28 ± 0.59	28.43 ± 0.40	28.21 ± 0.80	28.02 ± 0.43	27.93 ± 0.09
C16:1 n-10	0.73 ± 0.04^a^	0.60 ± 0.01^b^	0.73 ± 0.00^a^	0.62 ± 0.00^b^	0.67 ± 0.02	0.72 ± 0.03	0.67 ± 0.04	0.65 ± 0.01	0.69 ± 0.01	0.71 ± 0.01
C18:0	3.74 ± 0.30	3.99 ± 0.08	3.80 ± 0.16	3.83 ± 0.10	3.94 ± 0.05	3.89 ± 0.14	3.88 ± 0.18	4.01 ± 0.06	3.91 ± 0.08	4.00 ± 0.06
C18:1 *9c*	34.78 ± 0.88^a^	36.77 ± 0.58^b^	35.52 ± 0.36	36.91 ± 0.45	35.55 ± 0.57	35.33 ± 1.15	34.50 ± 1.77	36.14 ± 0.67	35.71 ± 0.57	35.97 ± 0.57
C18:1 *11c*	1.54 ± 0.10	1.62 ± 0.03	1.42 ± 0.27	1.59 ± 0.01	1.42 ± 0.23	1.65 ± 0.08	1.35 ± 0.18	1.43 ± 0.26	1.27 ± 0.04	1.42 ± 0.22
C18:2 *9c12c*	12.26 ± 0.34^a^	11.68 ± 0.24^b^	12.20 ± 0.12	12.06 ± 0.13	12.27 ± 0.01	12.12 ± 0.21	11.28 ± 0.76	11.47 ± 0.28	11.79 ± 0.34	11.87 ± 0.04
C20:0	0.38 ± 0.06^a^	0.45 ± 0.02^b^	0.41 ± 0.01	0.43 ± 0.03	0.40 ± 0.01	0.42 ± 0.02	0.38 ± 0.05	0.43 ± 0.01	0.42 ± 0.01	0.41 ± 0.01
C18:3 *9c12c15c*	2.81 ± 0.22^a^	2.28 ± 0.12^b^	2.88 ± 0.03^a^	2.49 ± 0.02^b^	2.84 ± 0.03	2.81 ± 0.05	2.81 ± 0.15	2.75 ± 0.18	2.74 ± 0.09	2.66 ± 0.05
Σ SFA	44.12 ± 3.15	43.68 ± 1.26	43.95 ± 0.43	43.11 ± 0.77	43.94 ± 1.35	44.15 ± 1.57	45.32 ± 2.19	43.74 ± 1.38	44.17 ± 1.21	44.03 ± 0.62
Σ SCSFA	8.43 ± 1.60	7.21 ± 0.27	7.95 ± 0.05	7.38 ± 0.07	7.63 ± 0.57	8.08 ± 0.59	8.79 ± 1.05	7.66 ± 0.35	8.31 ± 0.52	8.23 ± 0.40
Σ LCSFA	36.69 ± 1.55	36.47 ± 0.99	36.00 ± 0.38	35.73 ± 0.70	35.31 ± 0.78	36.08 ± 0.98	36.54 ± 1.14	36.08 ± 1.03	35.86 ± 0.69	35.80 ± 0.21
Σ MUFA	37.04 ± 2.52	38.99 ± 0.32	37.67 ± 0.53	39.12 ± 0.36	37.63 ± 0.83	37.70 ± 1.25	36.51 ± 1.99	38.21 ± 0.21	37.66 ± 0.62	38.09 ± 0.79
Σ PUFA	15.07 ± 0.61^a^	14.16 ± 0.32^b^	15.08 ± 0.17^a^	14.55 ± 0.14^b^	15.11 ± 0.04	14.93 ± 0.26	14.09 ± 0.91	14.22 ± 0.28	14.53 ± 0.43	14.68 ± 0.12

^a,b^ – values with different superscripts differ significantly (*p* ˂ 0.05) from the respective fresh group in row.

**Table 5:** Hedonic acceptability of muffins by assessors (n = 180).

Hedonic Value	Number of Assessors Making Hedonic Choices								
	Sucrose	Xylitol (0%)		Xylitol + 0.025% ChPE		Xylitol + 0.05% ChPE		Xylitol + 0.075% ChPE	
	Fresh	Fresh	Stored 3 Weeks	Fresh	Stored 3 Weeks	Fresh	Stored 3 Weeks	Fresh	Stored 3 Weeks
Like extremely - 9	8	18	13	13	10	9	6	0	0
Like very much - 8	34	54	48	55	48	46	41	15	11
Like moderately - 7	48	50	42	58	53	57	63	26	19
Like a little - 6	40	28	20	24	29	31	24	30	25
Neither like or dislike - 5	22	16	25	15	18	18	20	36	33
Dislike a little - 4	16	10	17	10	13	12	16	44	50
Dislike moderately - 3	8	4	10	5	8	5	7	17	22
Dislike a lot - 2	4	0	3	0	1	2	3	10	14
Dislike extremely - 1	0	0	2	0	0	0	0	2	6
Mean acceptability	6.26 ^a^	6.91 ^b^	6.37 ^A,*^	6.87 ^b^	6.59 ^B,*^	6.62 ^c^	6.43 ^A,*^	5.06 ^d^	4.64 ^C,*^
Range	2–9	3–9	1–9	3–9	2–9	2–9	2–9	1–8	1–8

Abbreviations: ^a,b,c,d^ - Values with different superscripts differ significantly (*p* < 0.05) from each other among fresh samples ^A, B, C^- Values with different superscripts differ significantly (*p* < 0.05) from each other among storage samples. Values with asterisk differ significantly (*p* < 0.05) from the respective fresh group in row. ChPE - Chokeberry polyphenol extract.
